# Premature Baldness

**Published:** 1885-01

**Authors:** 


### PREMATURE BALDNESS.

O. Lassar has continued his observations on the nature of premature baldness, and has further convinced himself of the communicability of at least the form associated with dandruff. When the hairs which fall off in such cases are collected, rubbed up with vaseline, and the ointment so made is rubbed among the fur of rabbits or white mice, baldness rapidly makes itself visible on the parts so treated. That this is not due to the vaseline was shown by anointing other animals with the vaseline alone, which produced no effect whatever. He considers that the disease is spread by hairdressers, who employ combs and brushes on their customers, one after another, without any regular cleansing of these articles after each time they are used. During frequent visits to the hairdressers it can scarcely fail that brushes are used which have been shortly before dressing the hair of one affected with so common a complaint as scaly baldness. Females, he thinks, are less often affected with this form of baldness, because the hairdresser more frequently attends to them at their own homes, and there uses their combs and brushes. In order to prevent as far as possible the commencement of alopecia prematura, the hair should be cut and dressed at home, and with one's own implements, and these thoroughly clean. When it has begun, the following mode of treatment is suggested: The scalp is to be daily well soaped with tar or fluid glycerine potash soap, which is to be rubbed in for fifteen minutes firmly. The head is then to be drenched with first warm water, and then gradually colder water. A two per cent corrosive sublimate lotion is next to be pretty freely applied. The head is then to be dried, and the roots of the hair are to have a one-half per cent. solution of naphthol in spirit rubbed into them. Finally, a pomade of 1½ to 2 per cent. of carbolic or salicylic oil is to be used on the head. This treatment has now in many cases brought the disease not only to a stand, but the hair has been to a considerable extent restored.

